# Short-Term Application of Alfalfa Green Manure Increases Maize Yield and Soil Fertility While Altering Microbial Communities in Karst Yellow Clay Soil

**DOI:** 10.3390/microorganisms13071445

**Published:** 2025-06-21

**Authors:** Xiaoye Gao, Shimei Yang, Yan He, Qiumei Zhao, Tao Zhang

**Affiliations:** 1 College of Eco-Environmental Engineering, Guizhou Minzu University, Guiyang 550025, China; gaoxiaoye1220@163.com (X.G.); heyan9622@163.com (Y.H.); 2 School of Earth System Science, Tianjin University, Tianjin 300072, China; 3 College of Resources and Environmental Engineering, Guizhou University, Guiyang 550025, China; gdyangshimei@163.com (S.Y.); gdzhaoqm@163.com (Q.Z.); 4 Guizhou Engineering Key Laboratory for Pollution Control and Resource Reuse Technology of Mountain Livestock Breeding, Institute of Rural Revitalization, Guizhou University, Guiyang 550025, China

**Keywords:** enzyme, soil quality index, bacteria, fungi, diversity, economic benefits

## Abstract

Green manure effectively improves soil nutrients and crop yields, yet its partial substitution for chemical nitrogen fertilizer (CF) in maize systems remains underexplored in ecologically fragile Karst landscapes. To assess the effect of alfalfa green manure on maize yield, soil nutrients, enzymes, and microorganisms, we conducted a two-year field experiment comprising eight treatments: four CF levels (100%, 80%, 60%, and 0% of recommended CF) applied alone or combined with alfalfa green manure (CF100, AL_CF100, CF80, AL_CF80, CF60, AL_CF60, CF0, AL_CF0). The results showed that maize grain yield decreased with the sole reduction of chemical N fertilizer. Compared to the CF100 treatment, the AL_CF100 and AL_CF80 treatments significantly increased grain yield by an average of 21.8% and 16.9%, respectively. Additionally, the AL_CF60 treatment maintained maize grain yield in 2020 and significantly increased it in 2021. The AL_CF100 treatment significantly enhanced soil available N (AN) content, while soil Olsen-P (SOP) content and soil quality index (SQI) were significantly improved in the AL_CF100, AL_CF80, and AL_CF60 treatments. Alfalfa green manure application had no significant effect on soil bacterial and fungal communities. However, the CF rates positively influenced the relative abundances of bacterial phyla (Bacteroidota, Myxococcota, and Patescibacteria) and genera (*Intrasporangium*, *Streptomyces*, and *Quadrisphaera*), as well as fungal genera (*Exophiala* and *Setophoma*). α-Diversity analysis revealed that partial substitution of CF with alfalfa green manure did not significantly affect soil bacterial diversity (Ace, Shannon, and Sobs indices) or richness (Chao value). In contrast, chemical N fertilizer rates significantly altered the β-diversity of both bacteria and fungi. The soil AN, AK, sucrase activity, and the relative abundances of Bacteroidota, *Streptomyces*, and *Instrasporangium* showed significant positive relationship with maize grain yield. This study demonstrates that substituting 20% CF with alfalfa green manure optimizes maize productivity while enhancing soil health in Karst agroecosystems.

## 1. Introduction

The Karst area in southwest China, one of the world’s largest continuous Karst regions (covering 55 × 10^6^ ha), is highly prone to soil degradation due to its complex soil– rock–flow system [1,2,3]. Yellow-clayed soil makes up 46.2% of the region’s soil, featuring weathering, erosion, and low AN and available phosphorus (AP) [4]. Inadequate cultivated area and poor soil quality, along with over-use of chemical fertilizers for higher yields, have led to visible rocky desertification, water eutrophication, and soil fertility loss [5,6,7,8]. Historically, over-application of chemical N fertilizers has boosted crop yields but also caused nitrogen loss, a major cause of water eutrophication and greenhouse gas emissions in recent decades [9].

Green manure is known to increase crop yield and soil nutrients while reducing the dependence on CF [10]. In particular, leguminous green manure coupled with reduction in chemical fertilizer can increase crop yield, soil nutrients, and enzymatic activities [11]. Utilization of milk vetch (*Astragalus sinicus* L.) to substitute 0–40% of chemical fertilizers increased rice yields by 6.3–15.6% [12]. Milk vetch incorporation with chemical fertilizer reductions of 20–40% significantly increased SOC, AN, and AP contents [11,13]. Most previous studies have focused on the effects of green manures on different soil indicators, and there is a lack of an evaluation of the effects on the integrated soil fertility in the short-term.

The SQI, obtained by integrating representative soil indicators, is a comprehensive and effective soil fertility assessment tool [14]. There is no universal soil indicator list for all ecosystems, and selected indicators must be management-sensitive [15]. Soil pH, SOC, TN, AN, AP, and AK contents are potential SQI indicators in field ecosystems [16]. High soil quality means high production without degradation. Thus, optimal fertilization should maintain high crop yield and soil fertility.

Soil microbes play an important role in nutrient cycling, organic matter decomposition, and crop nutrient utilization. It has been demonstrated that organic matter application can alter the soil microbial community by altering the habitat and providing nutrients for the soil microbiome [17,18,19]. Changes in soil pH, SOC, TN, AN, and enzyme activities by green manure amendment have been found to be important drivers of shifts in the soil microbial community [20,21,22]. Many studies have focused on legume–cereal rotation systems, which have been shown to increase plant-beneficial bacterial taxa [20,22,23]. Italian ryegrass (*Lolium multiflorum* L.) green manure increased the relative abundance of Proteobacteria, Bacteroidetes, Acidobacteria, and Gemmatimonadetes [19]. In a 31-year study on a rice system, Acinetobacter and Pseudomonas were involved in the plant-growth promotion by milk vetch, ryegrass, and rape (*Brassica campestris* L.) green manures [18]. Additionally, in a long-term study, the relative abundances of Mycobacterium and Pseudomonas, which are plant-beneficial bacteria, were higher in the hairy vetch–peanut rotation system [24]. In a 6-year field experiment, common vetch (*Vicia sativa* L.)–maize rotation increased the relative abundance of Actinobacteria and decreased that of Chloroflexi and Acidobacteria in a typical low-fertility farmland in arid land [25]. However, in Karst regions, studies related to the effects of green manure application on the soil microbial community are limited, and how the diversity and composition of the microbial community respond to the partial substitution of CF with alfalfa green manure is poorly understood.

Previous studies have mainly focused on the changes in crop yields, soil properties, and soil bacterial communities or cereal–green manure rotations based on the amount of traditional chemical fertilizer [18,26,27]. The application of leguminous green manure and reduction in CF contributed not only to nutrient cycling but also increased soil microbial activity [12,24]. In contrast, the response mechanism of soil properties, soil enzyme activities, and bacterial and fungal communities to leguminous green manure substitution with partial CF, and how soil nutrients and the bacterial and fungal community affect maize grain yield, remain unclear in yellow-clayed soil from Karst regions. This study aims to assess how alfalfa green manure affects maize yield, soil nutrients, enzymes, and microorganisms in maize cropping systems in the southwest Karst lands of China, and to explore the underlying mechanisms of these effects.

## 2. Materials and Methods

### 2.1. Experimental Site and Design

The field experiment was conducted in Tongmuzhai village, which is located in Pingba District, Guizhou, in southwest China (26°25′ N, 106°27′ E, 1244 m above sea level). The region is characterized by a sub-tropical humid monsoon climate with a mean annual temperature of 14.7 °C and rainfall of 1175 mm. The annual frost-free periods last 285 days. The soil is a typical yellow soil and classified as ultisol, with a pH of 5.28. The initial soil properties in the 0–20 cm soil layer were 20.27 g kg^−1^ SOC, 1.30 g kg^−1^ TN, and 1.24 g kg^−1^ TP.

The experiment utilized a randomized complete block design with three replicates, and each replicate plot was 3 m × 6 m. Four chemical N fertilizer levels, alone or in combination with alfalfa green manure, were applied to maize fields in 2020 and 2021, and the treatments were as follows: (1) CF100: chemical N fertilizer application with winter fallow; (2) AL_CF100: alfalfa grown in winter and incorporated as green manure combined with the chemical N fertilizer application; (3) CF80: 80% chemical N fertilizer application with winter fallow; (4) AL_CF80: alfalfa grown in winter and incorporated as green manure combined with 80% chemical N fertilizer application; (5) CF60: 60% chemical N fertilizer application with winter fallow; (6) AL_CF60: alfalfa grown in winter and incorporated as green manure combined with 60% chemical N fertilizer application; (7) CF0: no chemical N fertilizer application with winter fallow; (8) AL_CF0: alfalfa grown in winter and incorporated as green manure with no chemical N fertilizer application.

### 2.2. Field Management

Alfalfa was sown at a seeding rate of 27 kg ha^−1^ after the maize harvest in both 2020 and 2021. It was then cut into 5–10 cm pieces using a mower and incorporated into the soil at a depth of 0–20 cm with a rotary cultivator one week before maize seeding. The preceding crop was maize. No chemical fertilizers were applied during the alfalfa growth period. For maize cultivation, the traditional chemical fertilizer application rates were 244 kg N ha^−1^ and 145 kg P_2_O_5_ ha^−1^. All phosphorus fertilizers (ammonium dihydrogen phosphate, 11% N, 44% P_2_O_5_) were applied as basal fertilizers at maize seeding. Nitrogen fertilizer (urea, 46% N) was applied in two splits, 35% at the seedling stage and 55% before the tasseling stage.

Maize (cultivar: Sidanuo 41) was sown in bunches on 1 May 2020 and 4 May 2021. Each bunch contained 3–4 seeds, spaced approximately 60 cm apart. About two weeks after sowing, maize seedlings were thinned to two plants per bunch, maintaining 5 cm spacing between them, following local management practices. Maize was harvested above the ground on 27 August 2020 and 25 August 2021. Weeds were manually removed.

The dry alfalfa green manure contained 390.24 g kg^−1^ total carbon, 18.24 g kg^−1^ total nitrogen, 2.16 g kg^−1^ total phosphorus, and 7.02 g kg^−1^ total potassium. The incorporation rates of alfalfa green manure averaged 1380 kg ha^−1^ in 2020 and 2980 kg ha^−1^ in 2021.

### 2.3. Sampling and Analyzing

On the day of harvest, three soil cores along a diagonal collection pattern were collected and mixed as a replicate in each plot using a shovel to dig 20 cm soil profiles. Soil sampling points were selected in the central area between two maize bunches, ensuring they were outside root zones. Roots and visible impurities were manually removed from each soil sample. Each soil sample was then divided into three portions. One portion was passed through a 2 mm sieve and immediately frozen at −20 °C for the analysis of the soil microbial community, enzyme activities, and AN. Another portion was air-dried and passed through a 0.25 mm filter for the analysis of SOC, SOP, and soil available potassium (AK). It was also passed through a 0.15 mm filter for the analysis of soil total nitrogen (TN) and total phosphorus (TP) contents.

During the harvest, ten above-ground maize plants were randomly selected. The maize cobs were separated and dried at 60 °C, then threshed to measure the grain yield. Meanwhile, the straw was dried at 60 °C until it reached a constant weight for the measurement of straw biomass.

Soil pH was determined using a soil-to-water ratio of 1:2.5 with a glass electrode. SOC was measured using the dichromate oxidation method. Soil TN was determined using the sulfuric acid digestion–sodium salicylate method. Soil TP was measured using sulfuric acid digestion–molybdenum blue colorimetry. Soil AN (NO_3_^−^ and NH_4_^+^) content was extracted with 2 M KCl (soil:KCl (*V*/*V*) = 1:5) and measured using an automated procedure. SAP was extracted using 0.5 M NaHCO_3_ using the molybdenum antimony colorimetric method. Soil AK content was extracted with 1 M NH_4_OAC and measured using flame atomic absorption spectrophotometry. Soil catalase activity was determined using the titration method with permanganate, using hydrogen peroxide as the substrate [28]. Soil urease activity was determined using phenol-sodium hypochlorite colorimetry [28]. Soil acid phosphatase activity was determined using disodium phenyl phosphate colorimetry [29]. Soil sucrase activity was determined using 3,5-dinitrosalicylic acid colorimetry [30]. The above soil contents were calculated on a dry weight basis achieved by drying at 105 °C for 24 h.

### 2.4. Calculation of SQI and BCR

In this study, we selected six soil parameters to evaluate soil comprehensive fertility, including soil pH, SOC, TN, AN, AP, and AK, according to [16]. The calculation of the SQI involved the following steps: (1) calculation of the scores of soil parameters using a standard scoring function (Equation (1)) [31]; (2) calculation of the weight of each soil parameter by its commonality through principal component analysis [32]; (3) calculation of SQI based on the product of the score and the weight of each soil parameter (Equation (2)) [33], as follows:

(1) $$S=a/[1+{\left(\frac{x}{x_{0}}\right)}^{b}]$$

(2) $$\mathrm{SQI}=\sum_{i=1}^{n}S_{i}\times W_{i}$$

where S is the soil parameter score; a is the maximum score of soil parameter (a = 1); x is the actual measured value of the soil parameter; $x_{0}$ is the average value of each soil parameter; the values of b are −0.25 and 0.25, corresponding to the soil parameter “more is better” and “less is better”, respectively. $S_{i}$ is the soil parameter score; $W_{i}$ is the weight value of the soil parameter; *n* is the number of soil parameters.

Economic profitability of the different treatments was assessed based on the benefit-cost ratio (BCR), which is the ratio of the gross product in value (GPV) to the total cost of production according to the formula [34]:

BCR = GVP/COST(3)

The GPV is obtained by multiplying the gross physical product by the unit selling price of the product. The prices of ammonium dihydrogen phosphate and urea fertilizers are fixed by the “China Fertilizer Network”. The different labor costs are those applied to agricultural laborers in the study area (Table 1). If the BCR is greater than 1, then for every RMB 1 invested, the revenue generated is more than RMB 1, and the activity is considered economically profitable. On the other hand, if the BCR is less than 1, then for every RMB 1 invested, the income generated is less than RMB 1, and the activity is regarded as economically unprofitable.

### 2.5. Extraction of Soil DNA and PCR Amplification

The total soil DNA from a 0.5 g soil sample was extracted using a FastDNA^®^ Spin Kit for Soil (MP Biomedicals, Irvine, CA, USA) according to the manufacturer’s instructions. DNA concentration and purity were determined using a NanoDrop2000 spectrophotometer (Thermo Scientific, Waltham, MA, USA) and examined by running the sample on a 1% agarose gel. We amplified the V3–V4 region of the bacterial 16S rRNA with the universal forward primer 338F (5′-ACTCCTACGGGAGGCAGCAG-3′) and reverse primer 806R (5′-GGACTACHVGGGTWTCTAAT-3′) [21]. The fungal ITS gene region was amplified using the ITS1 (5′-CTTGGTCATTTAGAGGAAGTAA-3′) and ITS2 (5′-GCTGCGTTCTTCATCGATGC-3′) primers [17]. The thermal profile for amplification was as follows: initial denaturation at 95 °C for 3 min, denaturation at 95 °C for 30 s, annealing at 55 °C for 30 s, extension at 72 °C for 30 s, final extension at 72 °C for 10 min, for 27 cycles, according to Tks Gflex™ DNA Polymerase (R091S, Takara Bio Inc., Osaka, Japan). PCR amplicons were separated on 1% agarose gel and purified using an AxyPrep DNA Gel Extraction Kit (Axygen Biosciences, Union City, CA, USA). The amplicons were paired-end sequenced on an Illumina MiSeq platform (Majorbio, Shanghai, China).

### 2.6. Analysis of the Sequencing Data

Raw FASTQ files were de-multiplexed using an in-house Perl script and then quality-filtered using fastp version 0.19.6 and merged using FLASH version 1.2.7 [35] with the following criteria: (i) The reads were truncated at any site receiving an average quality score of <20 over a 50 bp sliding window, and truncated reads shorter than 50 bp or reads containing ambiguous characters were discarded; (ii) only overlapping sequences longer than 10 bp were assembled according to their overlapped sequence. The maximum mismatch ratio of the overlap region is 0.2. Reads that could not be assembled were discarded; (iii) samples were distinguished according to the barcode and primers, and the sequence direction was adjusted, exact barcode matching, two nucleotide mismatch in primer matching. Then the optimized sequences were clustered into operational taxonomic units (OTUs) using UPARSE 7.1 [36,37] with a 97% sequence similarity level. The most abundant sequence for each OTU was selected as a representative sequence. To minimize the effects of sequencing depth on alpha and beta diversity measures, the number of 16S rRNA gene sequences from each sample was rarefied to 20,000, which still yielded an average goods coverage of 99.09%, respectively.

### 2.7. Statistical Analysis

The least significant difference at a 5% confidence was used to identify differences in maize yield, soil properties, SQI, and soil enzyme activities among treatments using SAS v. 9.4 [38]. Pearson correlation analysis was used to analyze the effects of various indicators on maize yield using GraphPad Prism 10.0 software [39]. All figures were prepared using GraphPad Prism 10.0 software [39].

## 3. Results

### 3.1. Maize Production

**Figure 1:**
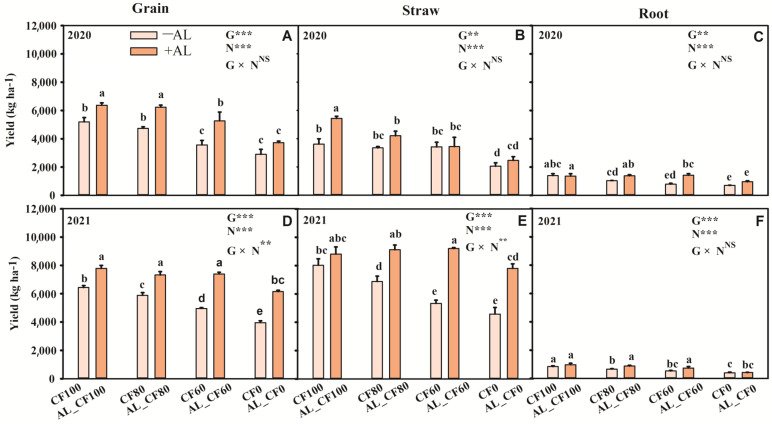
The yields of maize grain (**A**,**D**), straw (**B**,**E**), and root (**C**,**F**) under different treatments (*n* = 3). Vertical bars denote the standard deviation of the means. The different letters within the same year indicate significant differences between treatments at *p* < 0.05. Note: −AL and +AL indicate the treatments without the alfalfa green manure and with the alfalfa treatment, respectively. G, N, and G × N were the results of variance analysis of green manure, chemical N fertilizer, and their interaction, respectively. **, ***, and NS indicate significant at *p* < 0.01, *p* < 0.001, and insignificant differences, respectively.

Maize grain, straw, and root yields decreased as the sole chemical nitrogen (N) fertilizer application rate was reduced (Figure 1). Compared to CF100, maize grain yield decreased by an average of 27.20% and 41.42% in the CF60 and CF0 treatments, respectively (*p* < 0.05) (Figure 1A,D). The CF80 treatment maintained a grain yield comparable to CF100 in 2020 but significantly decreased by 8.56% in 2021 (*p* < 0.05). The grain yields were significantly increased by an average of 21.81% in the AL_CF100 treatment and 16.94% in the AL_CF80 treatment, respectively, compared to the CF100 treatment (*p* < 0.05). Compared to the treatments applying equal amounts of chemical N fertilizer, the application of alfalfa green manure significantly increased the grain yield by an average of 21.81%, 28.00%, and 48.58% in the AL_CF100, AL_CF80, and AL_CF60 treatments, respectively (*p* < 0.05). Compared to the CF100 treatment, the straw yields in the AL_CF100 and AL_CF80 treatments increased by an average of 30.23% and 15.15%, respectively (Figure 1B,E). No significant differences in root yields were observed between the CF100, AL_CF100 and AL_CF80 treatments (*p* > 0.05) (Figure 1C,F). The application of alfalfa green manure and chemical N fertilizer had significant effects on maize grain, straw, and root yields (*p* < 0.01), while their interactive effects on maize grain, straw, and root yields in 2020 and root yield in 2021 were not significant (*p* > 0.05).

### 3.2. Soil Property, Enzyme Activity, and SQI

The rates of chemical N fertilization had significant effects on each soil indicator (*p* < 0.05), except for soil TN, urease, phosphatase, and catalase activities, while alfalfa green manure primarily affected soil available nutrients, urease, and sucrase activities (*p* < 0.05) (Table 2 and Table 3). The pH was the highest in the 100% chemical N fertilization treatment and the lowest in the treatment without chemical N fertilization. Compared to the CF100 treatment, the AL_CF100 and AL_CF80 treatments significantly increased soil NH_4_^+^ content, and the AL_CF80 treatment significantly increased AP content (*p* < 0.05) (Table 2). Urease activity was significantly higher in the AL_CF100 treatment and lower in the AL treatment compared to the CF100 treatment (*p* < 0.05). The SQI values in the AL_CF100, AL_CF80, and AL_CF60 treatments were significantly higher than that in the CF100 treatment (*p* < 0.05), and the main effects of alfalfa green manure and chemical N fertilization on SQI were both highly significant (*p* < 0.001). The phosphatase activity showed a significant increase when combined with alfalfa green manure, particularly in the AL_CF100 and AL_CF80 treatments (*p* < 0.05). No significant differences in soil catalase activity were observed among the fertilization treatments (*p* > 0.05). Sucrase activity exhibited a significant decrease in the AL_CF80, AL_CF60, and AL treatments compared to the CF100 treatment (*p* < 0.05) (Table 3).

### 3.3. Soil Bacterial and Fungal Communities

**Figure 2:**
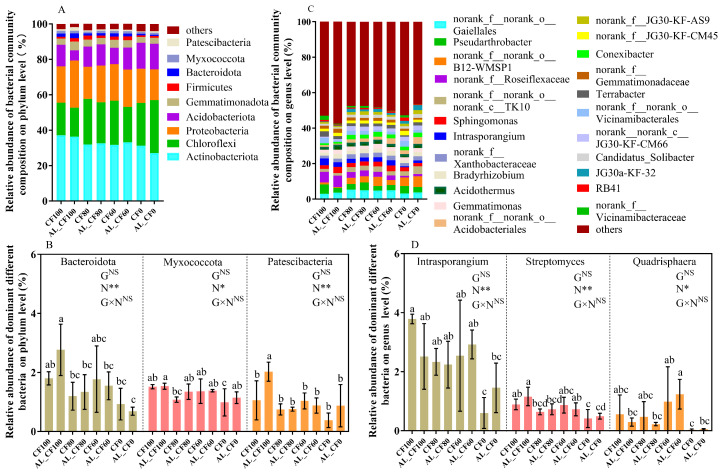
Bacterial community composition (**A**,**B**) and the relative abundances of dominant different bacteria (**C**,**D**) at the phylum and genus levels in 2021. Different letters indicate significant differences between treatments (*p* < 0.05). G, N, and G × N were the results of variance analysis of green manure, chemical N fertilization, and their interaction, respectively. *, **, and “NS” indicate significant at *p* < 0.05, *p* < 0.01, and insignificant differences, respectively.

**Figure 3:**
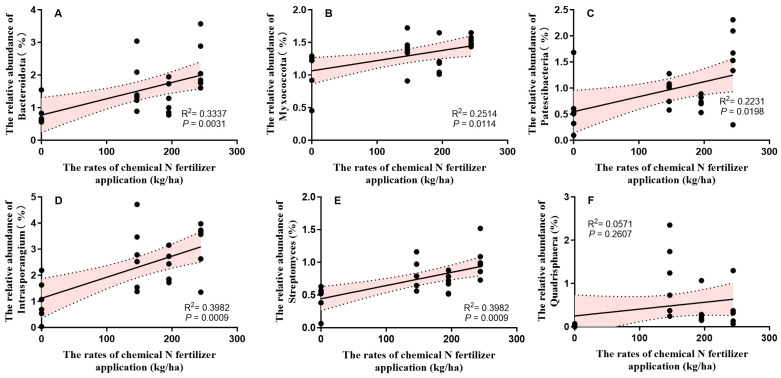
Linear relationships between the dominant bacteria at the phylum and genus levels and the rates of chemical N fertilizer application. (**A**) The relationship between the relative abundance of Bacteroidota and the rates of chemical N fertilizer application; (**B**) The relationship between the relative abundance of Myxococcota and the rates of chemical N fertilizer application; (**C**) The relationship between the relative abundance of Patescibacteria and the rates of chemical N fertilizer application; (**D**) The relationship between the relative abundance of *Intrasporangium* and the rates of chemical N fertilizer application; (**E**) The relationship between the relative abundance of *Streptomyces* and the rates of chemical N fertilizer application; (**F**) The relationship between the relative abundance of *Quadrisphaera* and the rates of chemical N fertilizer application. Pink area represents confidence interval.

At the phylum level, the bacterial community in each treatment was dominated by Actinobacteria, Chloroflexi, Proteobacteria, Acidobacteriota, and Gemmatimonadota, accounting for more than 90% of the total bacterial abundance (Figure 2A). Chemical N fertilization significantly altered the relative abundances of Bacteroidota, Myxococcota, and Patescibacteria (*p* < 0.05), with the highest levels observed in the AL_CF100 treatment. The relative abundance of Bacteroidota was the lowest in the AL_CF0 treatment, whereas Myxococcota and Patescibacteria exhibited their lowest relative abundances in the CF0 treatment (Figure 2B). Two-way ANOVA revealed that alfalfa green manure and its interaction with chemical N fertilization had no significant effects on the relative abundances of Bacteroidota, Myxococcota, and Patescibacteria (*p* > 0.05), whereas chemical N fertilization alone showed a significant effect (*p* < 0.05) (Figure 2C). The relative abundances of Bacteroidota, Myxococcota, and Patescibacteria were all significantly and positively correlated with chemical N fertilizer application (*p* < 0.05) (Figure 2A–C). The relative abundances of Bacteroidota, Myxococcota, and Patescibacteria were all significantly and positively correlated with chemical N fertilizer application rates (*p* < 0.05) (Figure 3A,B).

At the genus level, the relative abundances of the top 10 genera ranged from 0.27% to 11.39% (Figure 2C). The unclassified bacterium accounted for 5.24–7.81% of sequences across all samples. The relative abundances of *Intrasporangium*, *Streptomyces*, and *Quadrisphaera* differed significantly among fertilization treatments (*p* < 0.05) (Figure 2D). The relative abundance of *Intrasporangium* was the highest in the CF100 treatment, showing significantly higher levels than in the CF0 and AL_CF0 treatments (*p* < 0.05). The AL_CF100 treatment showed the highest relative abundance of *Streptomyces*, with values significantly greater than those observed in the CF80, AL_CF80, CF0, and AL_CF0 treatments (*p* < 0.05). The CF0 and AL_CF0 treatments showed the lowest relative abundances of *Intrasporangium*, *Streptomyces*, and *Quadrisphaera* (Figure 2D). Two-way ANOVA indicated a significant effect of chemical N fertilization (*p* < 0.05) but no significant effects of alfalfa green manure or its interaction with chemical N fertilization on these three genera (*p* > 0.05) (Figure 2D). The relative abundances of *Intrasporangium* and *Streptomyces* were both significantly and positively correlated with chemical nitrogen fertilizer application (*p* < 0.05) (Figure 3D,E). In contrast, there was no significant relationship between the relative abundance of *Quadrisphaera* and chemical nitrogen fertilizer application (*p* > 0.05) (Figure 3F).

**Figure 4:**
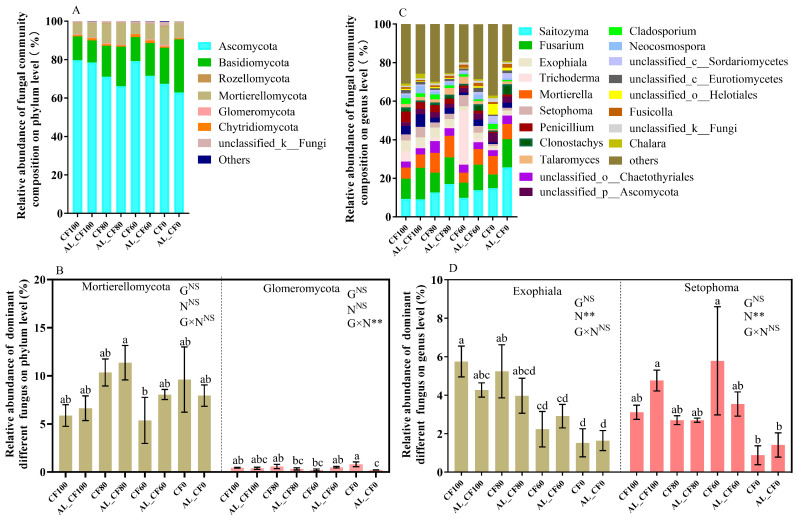
Fungal community composition (**A**,**B**) and the relative abundances of dominant different bacteria (**C**,**D**) at the phylum and genus levels in 2021. Different letters indicate significant differences between treatments (*p* < 0.05). G, N, and G × N were the results of variance analysis of green manure, chemical N fertilization, and their interaction, respectively. ** and “NS” indicate significant at *p* < 0.01 and insignificant differences, respectively.

At the phylum level, the relative abundances of Ascomycota and Basidiomycota collectively accounted for more than 80% of soil fungal communities, and unclassified fungi represented 0.16–4.10% across all samples (Figure 4A). Mortierellomycota reached its highest relative abundance in the AL_CF80 treatment, and Glomeromycota showed its lowest abundance in AL_CF0 (Figure 4C). Neither alfalfa green manure nor chemical N fertilization significantly affected the relative abundance of Mortierellomycota or Glomeromycota, whereas their interaction showed a significant effect on Glomeromycota abundance (*p* < 0.01).

**Figure 5:**
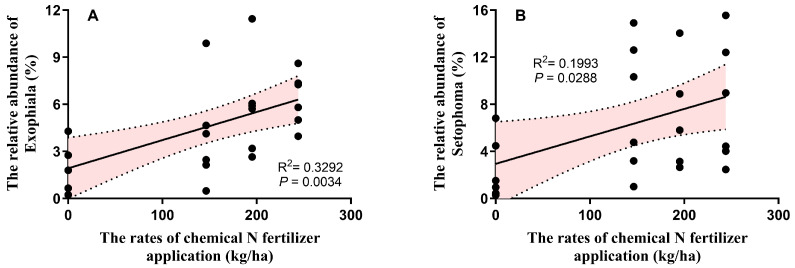
Linear relationships between the relative abundance of *Exophiala* (**A**) and *Setophoma* (**B**) and the rates of chemical N fertilizer application. Pink area represents confidence interval.

At the genus level, the relative abundances of the top 10 genera ranged from 0.81% to 25.68%, collectively accounting for 44.16–68.06% of the total soil fungal community (Figure 4B). *Exophiala* and *Setophoma* showed their lowest relative abundances in the CF0 treatment, with no significant differences observed among the CF100, AL_CF100, CF80, and AL_CF80 treatments (*p* > 0.05) (Figure 4D). Chemical N fertilizer significantly affected the relative abundances of *Exophiala* and *Setophoma* (*p* < 0.01), whereas alfalfa green manure alone and its interaction with chemical N fertilizer showed no significant effects (*p* > 0.05). Significant positive relationships were observed between the relative abundances of *Exophiala* and *Setophoma* and chemical N fertilizer application rates (Figure 5).

Three α-diversity indices (Ace, Chao, and Shannon) were used to assess soil bacterial and fungal community richness and diversity (Table 4). The AL_CF60 treatment showed the highest values for all three indices. Notably, the lowest bacterial Ace and Chao indices occurred in CF0, while the lowest fungal indices were observed in CF60.

**Figure 6:**
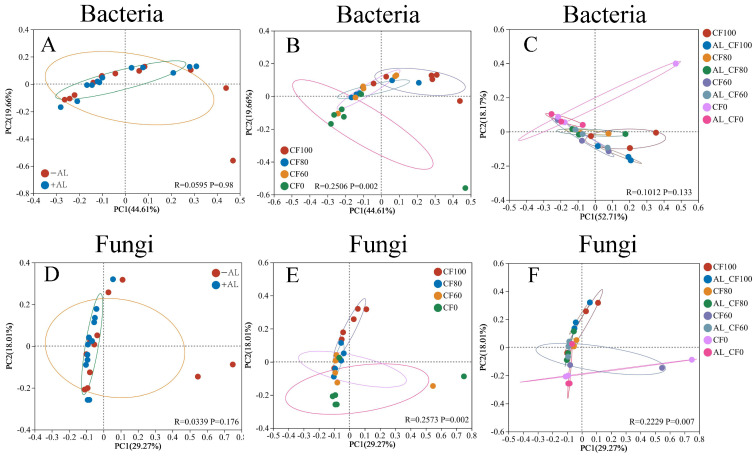
The bacterial (**A**–**C**) and fungal (**D**–**F**) community structure differences due to alfalfa green manure, chemical N fertilization rates, and treatment based on OTU evaluated by principal coordinates analysis (PCoA). Note: −AL and +AL indicate the treatments without the alfalfa green manure application and with the alfalfa green manure application, respectively. CF100, CF80, CF60, and CF0 represent the application of 100%, 80%, 60%, and 0% chemical N fertilizer, respectively.

β-Diversity analysis using PCoA revealed that neither alfalfa green manure application nor treatment significantly affected soil bacterial community composition (Figure 6A,C), while chemical N fertilization rates showed significant effects (Figure 6B). Alfalfa green manure application did not significantly alter fungal community composition compared to non-amended controls (Figure 6D). In contrast, chemical N fertilization rates and treatments significantly influenced fungal community composition (Figure 6E,F).

**Figure 7:**
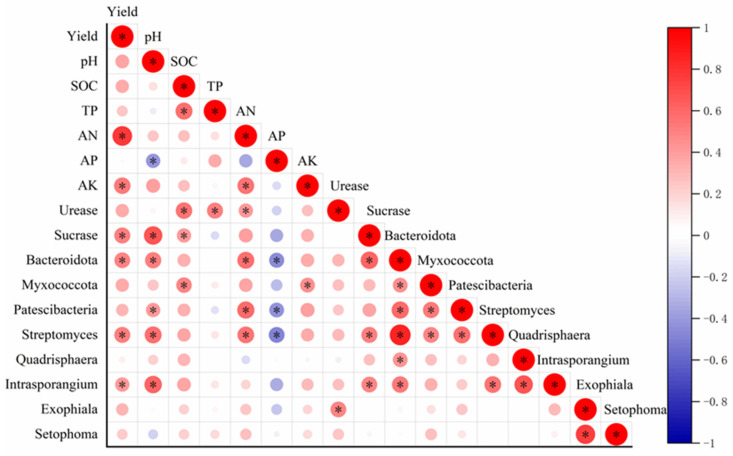
The correlation between yield and soil physicochemical properties, enzyme activities, and microorganisms. * indicates significant at *p* < 0.05.

Significant positive correlations were observed between maize grain yield and soil AN, AK, sucrase activity, the relative abundance of Bacteroidota, *Sreptomyces*, and *Intrasporangium* (*p* < 0.05) (Figure 7). Soil pH showed significant positive correlations with sucrase activity, the relative abundances of Bacteroidota, Patescibacteria, *Intrasporangium*, and *Streptomyces*. SOC was positively correlated with TP, urease activity, sucrase activity, and Myxococcota. Bacteroidota, Patescibacteria, and *Streptomyces* abundances were positively associated with soil AN. Conversely, soil AP demonstrated negative correlations with the relative abundances of Bacteroidota, Patescibacteria, and *Streptomyces* (*p* < 0.05).

### 3.4. Economic Profitability

**Figure 8:**
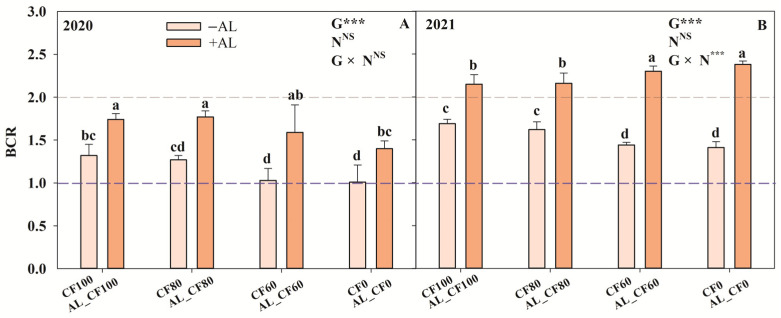
Benefit–cost ratio under different treatments in 2020 (**A**) and 2021 (**B**). Note: The different letters within the same year indicate significant differences between treatments at *p* < 0.05. −AL and +AL indicate the treatments without the alfalfa green manure and with the alfalfa treatment, respectively. G, N, and G × N were the results of variance analysis of green manure, chemical N fertilizer, and their interaction, respectively. *** and NS indicate significant at *p* < 0.001 and insignificant differences, respectively.

The application of alfalfa green manure significantly increased the BCR) (*p* < 0.001, whereas the application of chemical N fertilizer had no significant effect (*p* > 0.05) (Figure 8). Except for the treatment of CF0, the BCR values of all treatments ranged from 1 to 2 in 2020. The BCR values of all treatments involving the application of alfalfa green manure were above 2, while the BCR values of treatments with sole application of chemical N fertilizer were all under 2 in 2021. In 2020, the highest BCR values were observed in the AL_CF100 and AL_CF80 treatments, and in 2021, they were found in the AL_CF60 and AL_CF0 treatments.

## 4. Discussion

### 4.1. Partial Substitution of Chemical N Fertilizer with Alfalfa Green Manure on Maize Yield

The combined application of chemical fertilizer and green manure is widely recognized as an effective strategy for enhancing crop productivity [13,40,41]. This approach synergistically improves grain yield [10,27]. Our findings demonstrated that the integration of alfalfa green manure with 100% or 80% CF application rates significantly improved maize grain yield compared to sole applications of either treatment. These findings align with previous studies on Chinese milk vetch–maize rotation systems, where 20–60% CF substitution with green manure consistently improved rice yields over six growing seasons [10,13]. This can be attributed to the complementary nutrient supply; chemical N fertilizer provides readily available N for crop uptake, while alfalfa green manure contributes to gradual N mineralization, improving long-term N availability and reducing losses through leaching or volatilization [10,42]. Moreover, the synergistic increase in crop yield is inextricably linked to soil nutrient availability and beneficial microbial activity [42]. In this study, there were positive correlations between maize grain yield and soil AN (Figure 7). Previous studies demonstrated that applying milk vetch green manure alongside 20–40% chemical fertilizer reductions enhanced soil AN, the primary driver of increased rice yield [13,43]. The application of alfalfa green manure with CF reductions of 0–40% significantly increased soil NH_4_^+^ content (Table 1), consistent with Zhang et al. [16], who observed concurrent increases in soil AN and crop yield across 194 rapeseed green manure field trials. The soil-beneficial bacteria, Bacteroidota, *Streptomyces*, and *Intrasporangium* abundances were positively correlated with maize grain yield (Figure 7), which are effective indicators for soil fertility [44]. Similar results showed that hairy vetch green manure increased the abundance of Proteobacteria and potato yield [45], and milk vetch green manure increased the relative abundance of Actinobacteria and rice yield [22,46]. Therefore, alfalfa green manure substitution increases both soil nutrient availability and beneficial bacterial populations, with these combined effects playing important roles in enhancing crop yield.

### 4.2. Partial Substitution of CF with Alfalfa Green Manure on Soil Fertility and Enzyme Activities

Previous studies have demonstrated the large benefits of green manure application in partially replacing chemical fertilizers to improve soil nutrient availability [10]. In this study, the soil AN and TN contents were significantly enhanced in the AL_CF100 treatment, while soil TP and SOP contents clearly demonstrated superiority in the AL_CF80 treatment (Table 2). These findings align with Zhong et al. [21], who reported that long-term legume mulching in orchards increased soil AN and AP contents. Similarly, substituting 0–40% of chemical fertilizer with milk vetch green manure in rice systems enhanced soil AN and AP levels [10,22]. The dynamics of soil AN and AP contents are closely linked to N and P mineralization processes, which are positively correlated with total N and P concentrations but negatively associated with the C/N and C/P ratios of green manure [47,48]. Specifically, initial N mineralization by soil microbial biomass occurred when green manure with a C/N ratio below 25 decomposes in the soil [49], while P mineralization occurred when the C/P ratio is less than 112 [50]. The relatively low C/N and C/P ratios of alfalfa facilitate rapid decomposition, directly enhancing soil AN and AP availability. Additionally, soil nitrogen-fixing bacteria from the alfalfa rhizosphere can contribute to N cycling by converting atmospheric N_2_ into NH_4_^+^-N, which is subsequently oxidized to NO_3_^−^-N via nitrification [51].

In addition, alfalfa root exudates further promote nutrient solubilization [52,53]. These exudates contain organic acids capable of dissolving ferric phosphate to release P into the soil [54]. Specifically, organic anions enhance soil P availability through ligand exchange reactions that complex with Fe^3+^, Al^3+^, and Ca^2+^ cations, thereby mobilizing both organic and inorganic P pools [53]. Furthermore, alfalfa roots can secrete acid phosphatase that catalyze the mineralization of organic P into inorganic forms [53]. Notably, the soil Olsen-P content in the AL_100CF treatment showed a significant reduction compared to AL_80CF, AL_60CF, and AL treatments, likely attributable to enhanced P uptake efficiency by maize plants. In conclusion, these findings demonstrate that the integration of alfalfa green manure with 100% chemical N fertilizer application optimally improves soil AN content, while combinations with 0–80% chemical N fertilizer inputs more effectively enhance SOP content.

The combination of organic and inorganic fertilization application can enhance the SQI, a reliable indicator for evaluating soil fertility [12,16]. In this study, treatments combining alfalfa green manure with chemical fertilizer inputs demonstrated significantly higher SQI values compared to sole chemical fertilizer or alfalfa applications (Table 2). Soil AN, SOP, and pH were the key parameters driving SQI variations. Similar results showed that rapeseed green manure significantly increased SQI through improvements in soil TN, AN, and SOC [16]. This disparity likely stems from differential capacities to supply plant-available nutrients across fertilization regimes [16]. Our SQI calculation incorporated seven conventional soil fertility parameters: pH, SOC, TN, AN, TP, AP, and AK. Future research should expand this framework by integrating soil physical (e.g., aggregate stability, water retention) and biological indicators (e.g., microbial biomass, enzyme activities) to establish a more comprehensive soil quality assessment system.

Soil enzyme activities are crucial to soil biogeochemical cycles and act as important indicators of soil fertility levels [55]. Our findings demonstrated significant enhancements in urease (EC 3.5.1.5) and acid phosphatase (EC 3.1.3.2) activities under the AL_CF100 treatment (Table 3), consistent with previous observations in alfalfa-amended paddy systems [56], and a similar study applied milk vetch green manure in paddy fields [10]. The acid phosphatase catalyzes the hydrolysis of organic P compounds into plant-available orthophosphate ions, thereby mediating phosphorus mineralization from stable soil pools [57].

Soil urease rapidly hydrolyzes urea to ammonium and carbon dioxide and plays an important role in the N cycle [58]. In this study, the combined application of alfalfa green manure and a 100% CF rate significantly increased urease activity compared with the chemical fertilizer (Table 3). This result was in accordance with the finding of higher urease activity in hairy vetch green manure-amended soil compared with no green manure-amended soil [59]. Urease is widely distributed in microorganisms, plants, and animals [60]. The single application of alfalfa green manure decreased maize grain yield, soil chemical properties, SQI, and enzyme activities, which suggested that it is indispensable for the appropriate amount of CF application to increase crop yield and soil fertility.

### 4.3. The Effect of the Partial Substitution of CF with Alfalfa Green Manure on Soil Bacterial and Fungal Communities and Diversity

Soil microbial communities are sensitive to fertilization [23,61]. This study found that the application of alfalfa green manure had no significant effect on the composition of soil bacterial and fungal communities, whereas chemical N fertilizer application rates substantially altered these microbial assemblages. For bacteria, at the phylum level, the chemical N fertilizer rates had a significant effect on the relative abundances of Bacteroidota, Myxococcota, and Patescibacteria, showing a significant positive correlation with increasing chemical N fertilizer rates. These results support that bacteria with copiotrophs prefer the environment with high nutrient contents, while bacteria with oligotrophs likely thrive in low-nutrient conditions [62]. In previous studies, the relative abundances of Bacteroidota, Myxococcota, and Patescibacteria increased in high-N soils or in response to high chemical N fertilizer supply, but showed no significant response to organic substrate addition in the short-term [62]. Bacteroidota can suppress plant diseases, thereby reducing pathogen susceptibility, and are key contributors to soil organic phosphorus cycling [63]. Bacteroidota can decompose proteins and polysaccharides in the soil and convert them into small-molecule substances. These small-molecule substances not only provide carbon sources, nitrogen sources, and energy for the growth and metabolism of Bacteroidota itself, but also can be utilized by other soil microorganisms [64]. Myxococcota can break down complex organic matter such as lignin, cellulose, and proteins, releasing plant-available nutrients [65]. Patescibacteria may exert influence on the breakdown of organic matter, helping in the recycling of essential elements. Soil bacteria at the genus level are more sensitive to agricultural practices [66]. In this study, the relative abundances of *Intrasporangium*, *Streptomyces*, and *Quadrisphaera* showed an increasing trend as the rates of chemical N fertilizer application increased, while alfalfa green manure had no significant effect. *Intrasporangium*, *Streptomyces*, *Quadrisphaera* are copiotrophs, belonging to the Actinobacteria phylum. *Intrasporangium* participates in organic matter degradation, promoting soil carbon cycling. *Intrasporangium* mediates biological N_2_ fixation [67], while *Streptomyces* secretes proteases and chitinases to drive organic N mineralization, elevating NH_4_^+^ availability [68]. *Intrasporangium* and *Streptomyces* were positively correlated with soil available N content (Figure 7) increases in abundance as the rates of chemical N fertilizer application increased. *Streptomyces* are the most representative bacterial genera, and their metabolites have great potential to suppress pathogens and enhance plant growth in agricultural soils [69,70]. In addition, *Streptomyces* play an important function in nutrient turn over in soils [70].

Chemical N fertilizer rates had no significant effect on fungal phyla but showed a significant positive correlation with the relative abundances of *Exophiala* and *Setophoma* at the genus level, while alfalfa green manure incorporation had no significant effect on fungal communities in this study. Some *Exophiala* strains can convert insoluble P into soluble P by secreting acid phosphatase [71]. *Setophoma* exhibits plant-growth-promoting effects by enhancing growth parameters such as plant height and root length [72]. It also secretes a variety of extracellular enzymes to break down complex organic matter in the soil. This process promotes the release and cycling of elements such as carbon, nitrogen, and phosphorus in the soil, enabling these nutrients to be reused by other plants and microorganisms and maintaining the material balance of the soil ecosystem [73]. Therefore, the chemical N rates could enrich the relative abundances of beneficial bacteria and fungi, whereas alfalfa green manure had no significant effect on bacterial and fungal communities.

Neither chemical N fertilizer rates nor alfalfa green manure significantly affected the α-diversity of bacterial or fungal communities in this short-term study. However, chemical N fertilizer application rates significantly influenced β-diversity. Similar results reported chemical N fertilizer and chicken manure application for one season had no significant effect on soil bacterial and fungal community α-diversity [74]. These results contrast with observations from long-term green manure applications. A 31-year study showed that milk vetch green manure combined with chemical fertilizers reduced soil bacterial diversity and altered β-diversity in rice paddy fields [18]. Similarly, in the wheat–maize rotation system of the Hexi Corridor, seven years of common vetch green manure application decreased bacterial diversity but had no significant effect on fungal diversity [75]. In contrast, 14 years of green manure use in apple orchards enhanced fungal community diversity on China’s Loess Plateau [76]. These discrepancies may stem from variations in soil types, duration of green manure application, and cropping systems. Therefore, short-term application of green manure and chemical N fertilizer may alter the soil microbial community structure, composition, and β-diversity, but not significantly alter soil microbial community α-diversity.

## 5. Conclusions

These findings demonstrate that incorporating alfalfa green manure while reducing chemical N fertilizer application by 0–40% maintains or even increases maize yield. Additionally, applying 80–100% of the chemical N fertilizer rate in combination with alfalfa green manure enhances soil AN, SOP, and SQI, along with increased urease and acid phosphatase activities. Although the α-diversity of soil bacteria and fungi showed no significant changes, there was an increasing trend in certain beneficial bacteria and fungi at the phylum and genus levels as the chemical N fertilizer application rate increased. Economically, all combinations of chemical N fertilizer and alfalfa green manure were more profitable than sole application of chemical N fertilizer, and all their profits were significantly higher than CF100. In conclusion, alfalfa green manure can effectively reduce chemical N fertilizer use by at least 20% while improving maize yield and soil fertility in Karst regions of southwestern China. Future studies should further explore the role of functional soil microorganisms in long-term nutrient cycling.

## Figures and Tables

**Table 1:** Input and output costs in the economic analysis.

Item	Unit	Cost
Maize seed	CNY kg^−1^	50
Alfalfa	CNY kg^−1^	60
Ammonium dihydrogen phosphate fertilizer	CNY kg^−1^	4
Urea fertilizer	CNY kg^−1^	2.5
Tillage	CNY ha^−1^	1800
Sowing maize	CNY ha^−1^	1500
Sowing alfalfa	CNY ha^−1^	1000
Fertilizer application	CNY ha^−1^	120
Weeding	CNY ha^−1^	600
Harvesting	CNY ha^−1^	900
Ginning	CNY t^−1^	50
Maize grain	CNY kg^−1^	2.4
Maize straw	CNY kg^−1^	0.2

**Table 2:** Effects of different treatments on the soil properties and SQI (means ± SE).

Year	Treatments	pH	SOC (g/kg)	TN (g/kg)	TP (g/kg)	NH_4_^+^ (mg/kg)	NO_3_^−^ (mg/kg)	AP (mg/kg)	AK (mg/kg)	SQI
2020	CF100	6.1 ± 0.42 ab	34.22 ± 0.89 abc	1.42 ± 0.02 b	0.86 ± 0.11 bc	9.04 ± 1.14 c	10.11 ± 0.26 bc	10.7 ± 1.1 e	139.31 ± 3.57 ab	0.40 ± 0 d
	AL_CF100	6.24 ± 0.35 a	33.52 ± 0.59 bc	1.46 ± 0.15 ab	0.94 ± 0.05 abc	18.71 ± 1.07 a	10.95 ± 0.2 abc	14.62 ± 1.04 de	152.7 ± 7.8 ab	0.41 ± 0 bc
	CF80	5.62 ± 0.14 abc	37.27 ± 0.6 a	1.62 ± 0.16 ab	1.05 ± 0.07 ab	9.02 ± 0.13 c	12.76 ± 1.52 a	19.2 ± 0.83 cd	133.07 ± 15.3 b	0.41 ± 0 b
	AL_CF80	5.74 ± 0.3 abc	36.01 ± 1.07 ab	1.8 ± 0.05 a	1.11 ± 0.03 a	20.75 ± 1.8 a	11.7 ± 0.23 ab	20.27 ± 1.39 bc	105.87 ± 12.91 c	0.42 ± 0 a
	CF60	5.59 ± 0.24 abc	36.91 ± 2 a	1.55 ± 0.12 ab	0.94 ± 0.09 abc	7.51 ± 0.4 c	9.69 ± 1.1 bc	17.99 ± 0.94 cd	163.24 ± 3.2 a	0.41 ± 0 bc
	AL_CF60	5.7 ± 0.11 abc	35.04 ± 0.31 ab	1.46 ± 0.23 ab	1.09 ± 0.05 a	14.81 ± 0.5 b	8.58 ± 1.13 c	28.44 ± 3 a	93.52 ± 6.08 cd	0.42 ± 0 a
	CF0	5.19 ± 0.13 c	29.8 ± 1.43 d	1.35 ± 0.04 b	0.8 ± 0.08 c	6.41 ± 0.55 c	9.58 ± 0.27 bc	19.8 ± 1.59 bcd	88.34 ± 7.97 cd	0.41 ± 0 cd
	AL_CF0	5.42 ± 0.11 bc	31.02 ± 1.11 cd	1.43 ± 0.02 b	0.91 ± 0.05 abc	8.52 ± 1.01 c	9.39 ± 0.51 bc	23.63 ± 2.62 ab	76.41 ± 5.06 d	0.41 ± 0 bc
	G	NS	NS	NS	NS	***	NS	**	**	***
	N	*	***	NS	*	***	**	***	***	***
	G × N	NS	NS	NS	NS	***	NS	NS	**	NS
2021	CF100	6.45 ± 0.24 a	39.42 ± 1.01 bcd	1.41 ± 0.1 abc	0.84 ± 0.05 b	11.14 ± 0.25 cd	10.89 ± 0.57 ab	7.42 ± 0.99 c	135.07 ± 18.13 b	0.41 ± 0 d
	AL_CF100	6.14 ± 0.32 ab	42.23 ± 0.65 ab	1.8 ± 0.19 a	0.98 ± 0.07 ab	30.85 ± 0.58 a	11.77 ± 0.15 a	10.52 ± 0.92 c	146.78 ± 2.77 b	0.43 ± 0 a
	CF80	5.78 ± 0.24 ab	44.11 ± 0.71 a	1.61 ± 0.07 ab	1.12 ± 0.11 a	9.71 ± 0.35 d	8.75 ± 0.47 c	18.89 ± 1.24 b	61.9 ± 5.58 d	0.42 ± 0 abc
	AL_CF80	5.86 ± 0.37 ab	42.93 ± 0.96 ab	1.57 ± 0.1 ab	1.17 ± 0.01 a	16.24 ± 0.3 b	9.36 ± 0.15 bc	27.19 ± 1.44 a	177.33 ± 8.2 a	0.43 ± 0 a
	CF60	5.87 ± 0.23 ab	41.39 ± 2.49 abc	1.44 ± 0.05 abc	1.03 ± 0.14 ab	6.59 ± 0.56 e	9.19 ± 1.47 bc	19.86 ± 1.92 b	81.78 ± 10.07 cd	0.42 ± 0 cd
	AL_CF60	5.93 ± 0.11 b	41.73 ± 0.98 ab	1.53 ± 0.13 abc	1.00 ± 0.08 ab	12.77 ± 1.21 c	8.33 ± 0.93 c	27.13 ± 1.09 a	93.74 ± 11.96 c	0.42 ± 0 ab
	CF0	5.51 ± 0.18 ab	37.4 ± 0.6 cd	1.29 ± 0.11 bc	0.82 ± 0.06 b	3.27 ± 0.36 f	9.9 ± 0.59 abc	20.43 ± 1.92 b	92.26 ± 5.99 c	0.41 ± 0 d
	AL_CF0	5.74 ± 0.12 bc	35.52 ± 2.15 d	1.13 ± 0.26 c	0.93 ± 0.06 ab	11.36 ± 1.12 cd	9.79 ± 0.16 abc	22.25 ± 0.26 b	73.87 ± 5.28 cd	0.42 ± 0 cd
	G	NS	NS	NS	NS	***	NS	***	**	***
	N	NS	***	*	*	***	**	***	***	*
	G × N	NS	NS	NS	NS	***	NS	NS	**	NS

Note: Values with different letters within a column in the same year were statistically different at *p* < 0.05; G, N, and G × N were the results of variance analysis of green manure, chemical N fertilizer, and their interaction, respectively. *, **, ***, and NS indicate significant at *p* < 0.05, *p* < 0.01, *p* < 0.001, and insignificant differences, respectively.

**Table 3:** Effects of different treatments on the soil enzyme activities (means ± SE).

Treatments	Urease (mg/g)		Phosphatase (mg/g)		Sucrase (mg/g)		Catalase (mg/g)	
	2020	2021	2020	2021	2020	2021	2020	2021
CF100	0.50 ± 0.03 b	0.46 ± 0.05 ab	1.87 ± 0.24 b	5.35 ± 0.51 ab	6.27 ± 0.77 a	5.07 ± 0.3 a	2.04 ± 0.25 a	2.54 ± 0.05 a
AL_CF100	0.37 ± 0.03 b	0.49 ± 0.03 a	2.27 ± 0.39 ab	5.94 ± 0.45 a	7.08 ± 0.64 a	5.27 ± 0.35 a	2.35 ± 0.12 a	2.77 ± 0.26 a
CF80	0.67 ± 0.11 a	0.50 ± 0.07 a	2.75 ± 0.42 a	5.58 ± 0.43 ab	5.66 ± 0.83 ab	4.07 ± 1.49 ab	2.33 ± 0.31 a	2.52 ± 0.08 a
AL_CF80	0.47 ± 0.08 b	0.48 ± 0.03 ab	2.21 ± 0.09 ab	5.95 ± 0.18 a	6.41 ± 0.75 a	3.47 ± 0.41 ab	2.13 ± 0.32 a	2.5 ± 0.18 a
CF60	0.41 ± 0.01 b	0.40 ± 0.03 ab	2.03 ± 0.05 b	5.05 ± 0.4 ab	3.75 ± 0.49 cd	3.25 ± 0.91 ab	1.99 ± 0.04 a	2.81 ± 0.07 a
AL_CF60	0.44 ± 0.04 b	0.39 ± 0.02 ab	1.99 ± 0.06 b	5.07 ± 0.15 ab	5.98 ± 0.44 ab	4.51 ± 0.95 ab	2.02 ± 0.02 a	2.59 ± 0.14 a
CF0	0.52 ± 0.04 ab	0.35 ± 0.02 b	1.71 ± 0.06 b	4.72 ± 0.12 b	1.83 ± 0.57 d	2.35 ± 0.42 b	1.99 ± 0.01 a	2.36 ± 0.1 a
AL_CF0	0.46 ± 0.03 b	0.38 ± 0.07 ab	1.96 ± 0 b	4.53 ± 0.37 b	4.23 ± 0.66 bc	2.64 ± 0.73 b	2.02 ± 0.1 a	2.57 ± 0.02 a
G	*	NS	NS	NS	**	NS	NS	NS
N	NS	*	NS	*	***	***	NS	NS
G × N	NS	NS	NS	NS	NS	NS	NS	NS

Note: Values with different letters within a column were statistically different at *p* < 0.05; G, N, and G × N were the results of variance analysis of green manure, chemical N fertilizer, and their interaction, respectively. *, **, ***, and NS indicate significant at *p* < 0.05, *p* < 0.01, *p* < 0.001, and insignificant differences, respectively.

**Table 4:** Impacts of fertilization on soil bacterial and fungal Ace, Chao, and Shannon indexes (means ± SE).

Treatments	Bacteria			Fungi		
	Ace	Chao	Shannon	Ace	Chao	Shannon
CF100	3335.3 ± 218.19 abc	2991.21 ± 77.09 b	6.17 ± 0.06 bc	1057.49 ± 68.37 ab	1058.92 ± 71.15 ab	4.10 ± 0.22 a
AL_CF100	3029.11 ± 189.29 bc	2941.4 ± 70.75 b	6.12 ± 0.03 c	954.17 ± 51.41 abc	946.13 ± 49.73 abc	3.98 ± 0.1 a
CF80	3177.14 ± 89.43 abc	2927.21 ± 204.77 b	6.16 ± 0.07 bc	941.12 ± 0.78 abc	937.89 ± 2.38 abc	4.06 ± 0.05 a
AL_CF80	3241.27 ± 146.9 abc	2952.03 ± 142.83 b	6.14 ± 0.03 c	873.74 ± 52.01 abc	870.89 ± 57.23 abc	3.80 ± 0.19 a
CF60	3389.01 ± 127.28 abc	3301.92 ± 247.59 ab	6.27 ± 0.15 abc	755.47 ± 141.27 c	754.6 ± 145.97 c	3.06 ± 0.95 a
AL_CF60	3575.97 ± 208.63 a	3574.7 ± 192.35 a	6.48 ± 0.1 a	1068.54 ± 40.01 a	1067.27 ± 35.06 a	4.13 ± 0.11 a
CF0	2909.75 ± 200.33 c	2938.62 ± 188.12 b	6.27 ± 0.07 abc	835.95 ± 119.72 bc	833.81 ± 116.65 bc	3.92 ± 0.27 a
AL_CF0	3516.28 ± 34.86 ab	3522.07 ± 69.95 a	6.40 ± 0.08 ab	875.33 ± 29.53 abc	873.02 ± 25.31 abc	3.40 ± 0.13 a

Note: Values with different letters within a column in the same year were statistically different at *p* < 0.05.

## Data Availability

The datasets generated and analyzed during the current study are available from the corresponding author upon reasonable request.
